# Comparing Radiation Dose of Cerebral Angiography Using Conventional and High kV Techniques: A Retrospective Study on Intracranial Aneurysm Patients and a Phantom Study

**DOI:** 10.3390/tomography9020050

**Published:** 2023-03-08

**Authors:** Woranan Kirisattayakul, Panuwat Pattum, Waranon Munkong, Thawatchai Prabsattroo, Chonnatcha Khottapat, Tanyalak Chomkhunthod, Vithit Pungkun

**Affiliations:** 1 Department of Radiology, Faculty of Medicine, Khon Kaen University, Khon Kaen 40002, Thailand; 2 Office of Atoms for Peace, Ministry of Higher Education, Science, Research and Innovation, Bangkok 10900, Thailand

**Keywords:** high kV, cerebral angiography, aneurysm diagnosis, radiation dose, optically stimulating luminescence dosimeter

## Abstract

Evaluation of patient radiation dose after the implementation of a high kV technique during a cerebral angiographic procedure is an important issue. This study aimed to determine and compare the patient radiation dose of intracranial aneurysm patients undergoing cerebral angiography using the conventional and high kV techniques in a retrospective study and a phantom study. A total of 122 cases (61 cases with conventional technique and 61 cases with high kV technique) of intracranial aneurysm patients, who underwent cerebral angiographic procedure and met the inclusion criteria, were recruited. The radiation dose and the angiographic exposure parameters were reviewed retrospectively. The radiation dose in the phantom study was conducted using nanoDot^TM^ optically stimulating luminescence (OSLD), which were placed on the scalp of the head phantom, the back of the neck, and the phantom skin at the position of the eyes. The standard cerebral angiographic procedure using the conventional and high kV techniques was performed following the standard protocol. The results showed that the high kV technique significantly reduced patient radiation dose and phantom skin dose. This study confirms that the implementation of a high kV technique in routine cerebral angiography for aneurysm diagnosis provides an effective reduction in radiation dose. Further investigation of radiation dose in other interventional neuroradiology procedures, particularly embolization procedure, should be performed.

## 1. Introduction

Intracranial aneurysm is the main cause of subarachnoid hemorrhage (SAH), with a mortality rate of approximately 40%, and a leading cause of disability [1,2,3]. The worldwide incidence of SAH is 6.67 per 100,000 people [4]. Cerebral angiography using bi-plane digital subtraction angiography (DSA) with two-dimensional angiography (2DA) and three-dimensional rotational angiography (3DRA) is the gold standard for the diagnosis of intracranial aneurysm [5]. Although cerebral angiography provides a high effectiveness in this issue, the exposure of patients to high radiation dose is also mentioned [6]. A high radiation dose may cause deterministic skin injury and increases the risk of stochastic or cancer effect [7]. Therefore, the optimization of angiographic technique and radiation dose has gained much attention.

Numerous studies have demonstrated the effectiveness of optimization techniques in cerebral angiographic procedures [8,9,10,11]. According to the differences in each DSA unit, the work process, and the factors affecting radiation dose, proper optimization technique should be of concern. A previous study of our institute revealed that the radiation dose of the cerebral angiographic procedure for intracranial aneurysm diagnosis gradually increased. Moreover, the typical value of radiation dose from this procedure was also found to be higher than the values used in other institutes. The main factor affecting radiation dose was a reduction in kV in 2DA, which in turn increased the mAs value and radiation dose [12]. The use of a high kV technique in 2DA had been performed and implemented since 1 July 2019 to reduce radiation dose. The clinical image quality had been approved by the interventional neuroradiologist. However, the monitoring and investigation of patient radiation dose, which is regarded as an important step for dose optimization process, [13] had not been performed. This study aimed to determine and compare the patient radiation dose of intracranial aneurysm patients undergoing cerebral angiography using conventional and high kV techniques in a retrospective study and a phantom study.

## 2. Materials and Methods

### 2.1. Study Design and Ethical Consideration

This study used a comparative study design to determine radiation dose from a retrospective study by collecting patients’ radiation dose data, and a phantom study was also performed. This study was conducted in accordance with the Declaration of Helsinki. All methods and data collection of this study were approved by our institutional Ethics Committee for Human Research (HE641276). The retrospective review was conducted from 9 January 2018 to 29 December 2020. Data collection of patient radiation dose from cerebral angiogram using the conventional technique was performed from 9 January 2018 to 30 June 2019, and data collection for the high kV technique was performed from 1 July 2019 to 29 December 2020. The phantom study was conducted to compare phantom skin dose from cerebral angiography using the nanoDot™ OSLD (Landauer Inc., Glenwood, Illinois, USA).

### 2.2. Sample Size Calculation and Participant Selection

All participants involved in this study were SAH patients who underwent cerebral angiography after an intracranial aneurysm diagnosis. They were divided into 2 groups in accordance with their angiographic techniques, namely the conventional technique and the high kV technique. The sample size calculation was conducted by a biostatistician, and the data used for the calculation were from previous studies [12,14]. The sample size was 61 participants per group for a power of 0.8 and an α of 0.05.

The inclusion criteria were as follows: (1) SAH patients of both genders who were aged 18 years old and above; (2) angiographic procedure was performed by Dr. Waranon Munkong; (3) cerebral angiography was performed in 3 main vessels (left and right internal carotid artery, and vertebral artery) with the 2DA technique (6 exposures), 3DRA technique (6 exposures), and fluoroscopic technique; and 4) the use of a small focal spot size. Patients who met the following exclusion criteria were excluded: (1) aged under 18; (2) angiographic procedure was performed by other doctors; (3) angiographic procedure was performed on more or less than 3 vessels; and (4) lack of some angiographic techniques. A total of 122 cases were recruited into this study (61.48% female and 38.52% male, 56.8 ± 13.1 years old). There were 38 females and 23 males in the conventional technique group, while 37 females and 24 males were in the high kV technique group.

### 2.3. Cerebral Angiographic Protocol

All procedures were performed using a flat-panel detector and the vascular intervention system Artis Zee biplane (Siemens Medical Solutions, Munich, Germany), which is annually checked for quality control and verified for the dose area product meter by the Department of Medical Sciences, Ministry of Public Health. The standard protocol of cerebral angiography for intracranial aneurysm diagnosis in our institute consists of 2DA or acquisition angiography in the posteroanterior (PA) view along with the lateral view, 3DRA, and fluoroscopy. Each technique provides a distinct benefit for the diagnosis of intracranial aneurysm. The desirable kV of each technique was set by a product specialist of Siemens Healthineers Limited (Bangkok, Thailand) under a local service mode. The kV of the conventional technique was set at 70 kV, while for the high kV technique, it was 90 kV. Although kV was set for a specific value, it could be deviated to the range of the desirable kV, which was controlled by an automatic exposure control (AEC), depending on the thickness of the patient’s head. Moreover, other exposure parameters, including mA, exposure time, Cu filter thickness, were also varied and controlled by the AEC. The fluoroscopy was set at 10 pulses per second (p/s) for both techniques, which was the optimal pulse for fluoroscopy in clinical performance. In addition, the 3DRA technique was not modified and was kept constant in both techniques according to the recommendation of a product specialist.

### 2.4. Collection of Cerebral Angiographic Exposure Parameters and Patient Radiation Dose

A review of patient radiation dose was performed and collected from the picture archiving communication system by using the patient database of the Interventional Neuroradiology unit. The data were recorded in Microsoft Excel as follows: 2DA exposure parameters (kV, mAs, acquisition time, and additional Cu filter thickness), 3DRA exposure parameters (kV and mAs), total number of angiographic images (2DA images + 3DRA images), fluoroscopy time, dose area product (DAP; µGy·m^2^) and reference air kerma (RAK; mGy) of the 2DA, 3DRA, fluoroscopy, and the total DAP and RAK (2DA + 3DRA + fluoroscopy).

### 2.5. Phantom Study Protocol and Setting

**Figure 1:**
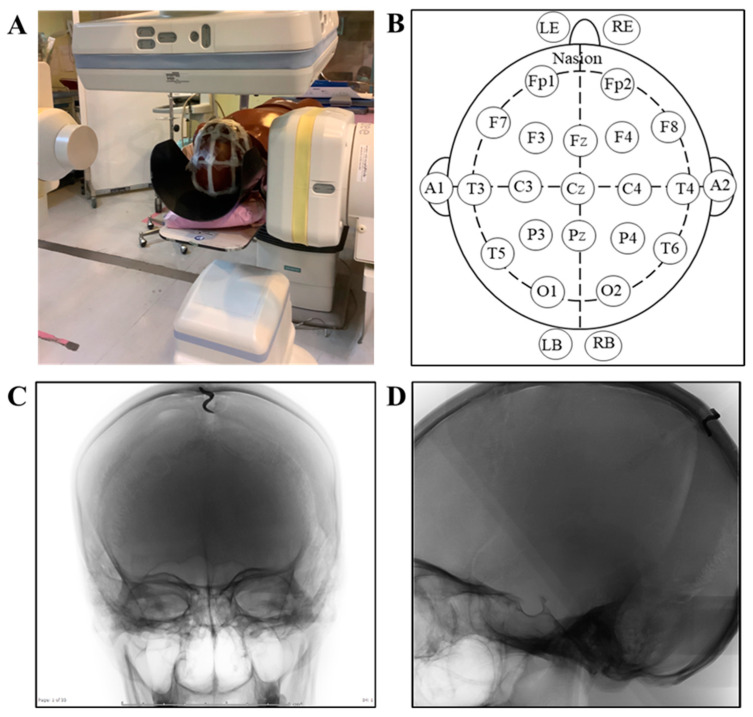
Phantom setting to evaluate radiation dose using nanoDot™ OSLDs: (**A**) phantom setting during the cerebral angiographic procedure; (**B**) the top view of the head demonstrates the location of the nanoDot™ OSLDs placed on the head phantom according to the 10/20 lead system (pre-frontal: Fp1, Fp2; frontal: F7, F3, Fz, F4, F8; temporal: T3, T5, T4, T6; parietal: P3, Pz, P4; occipital: O1, O2; center: C3, Cz, C4; and Auricle: A1, A2), on the eye lens (LE: left eye lens; RE: Right eye lens) and on the back of the neck on the left side (LB) and the right side (RB); (**C**) field of view of skull phantom from PA view; and (**D**) field of view of skull phantom from lateral view.

The bi-plane DSA was set up in the same condition for both the conventional and high kV techniques. The cerebral angiographic parameters used in the phantom study are shown in Table 1. The setting was based on the appropriate image size of the skull presented on the monitor and covered the whole skull during rotation in the same pattern as in a real situation. The setting of the angiographic protocol was performed by a product specialist of Siemens Healthineers Limited (Thailand). Then, twenty-one nanoDot™ OSLDs were placed on the scalp of the head phantom. The location of placement was based on the 10–20 system of electroencephalogram measurement for the convenience of location specification. Moreover, two nanoDot™ OSLDs were also placed on the back of the neck (right back, RB; left back, LB), which was the entrance point of radiation beam, and two of these were also placed on the phantom skin at the location of the eyes to represent the eye lens (left eye lens, LE; right eye lens, RE). Then, the head phantom was placed in a head support, and the body phantom was placed next to the head phantom lying on the angiographic table. The phantom study setting is shown in Figure 1. The cerebral angiographic procedures and radiation measurements were conducted 3 times in each technique. The data for the dose report, including DAP and RAK of the 2DA, 3DRA, and fluoroscopy, total DAP (2DA + 3DRA + fluoroscopy), and total RAK (2DA + 3DRA + fluoroscopy), were also recorded. To evaluate the radiation dose, the nanoDot™ OSLDs were sent to the Personal Dosimetry Laboratory at the Office of Atoms for Peace (OAP).

### 2.6. Evaluation of Phantom Radiation Dose

The NanoDot™ OSLDs were read by a microStar reader (Landauer Inc., Glenwood, IL, USA) to evaluate the radiation dose in the phantom study. The internal algorithm and calibration dosimeters were used to calculate the photon radiation dose in the nanoDot™ OSLDs, which can be traceable to the National Metrology Institute of Japan, Japan. The control and standard dosimeters obtained from the Secondary Standard Dosimetry Laboratory—OAP were included in the reading process for quality control. All nanoDot™ OSLDs were read twice, and the radiation dose in each location was averaged. The phantom skin doses were expressed as mean value in mGy unit.

### 2.7. Statistical Analysis

Statistical analysis was performed using SPSS version 21. All data were tested for normality using the Shapiro–Wilk test, where *p*-value > 0.05 represented a normal distribution. Independent sample *t*-test (normal distribution of data set) and Mann–Whitney U test (abnormal distribution of data set) were performed to analyze significant differences in angiographic exposure parameters between the groups. To analyze patient radiation dose, Quade’s ANCOVA was used to analyze the 2DA DAP, 2DA RAK, total DAP, and total RAK, while using the number of images and acquisition time as covariates. The DAP and RAK of the 3DRA and fluoroscopy were analyzed by using independent sample *t*-test and Mann–Whitney U test, respectively. A comparison of radiation doses from the phantom study was performed using independent sample *t*-test. The angiographic exposure parameters were presented as mean ± SD and median, while the patient radiation dose was presented as median. The data of the phantom study was expressed as mean ± SD. A *p*-value <0.05 was regarded as demonstrating significant difference.

## 3. Results

### 3.1. Effects of the High kV Technique on Cerebral Angiographic Exposure Parameters

The cerebral angiographic techniques are shown in Table 2. It was found that the kV values of the high kV technique from the 2DA in both PA and lateral view showed a significant difference from the conventional technique (*p*-value < 0.001 for all). The elevation of kV in the high kV technique resulted in a significant reduction in mAs (*p*-value < 0.001 and 0.05, respectively). In addition, the elevation of kV also led to a significant increase in the Cu filter thickness in both PA and lateral view (*p*-value < 0.001). The kV and mAs in the 3DRA technique and the fluoroscopic time did not bring about any significant difference. Interestingly, the 2DA acquisition time (sec) and the total number of images in the high kV technique were significantly reduced (*p*-value < 0.05 for all). Based on the basic knowledge, these two parameters are the important factors that affect radiation dose. Therefore, they were kept as covariates for further analysis of radiation dose parameters.

### 3.2. Effects of the High kV Technique on Patient Radiation Dose

**Figure 2:**
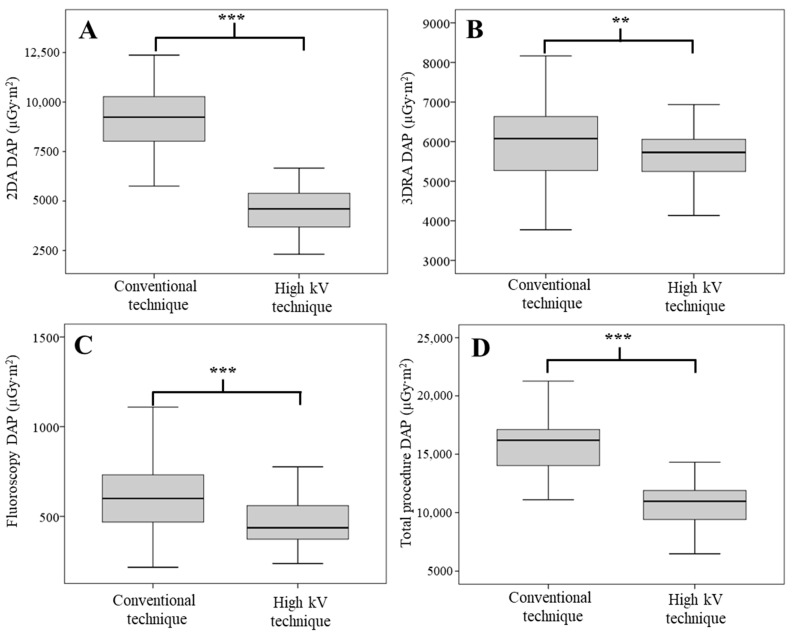
DAP from the 2DA technique (**A**), 3DRA technique (**B**), fluoroscopic technique (**C**), and total DAP (**D**). ** and *** indicate significant differences with *p*-value < 0.01 and 0.001, respectively.

**Figure 3:**
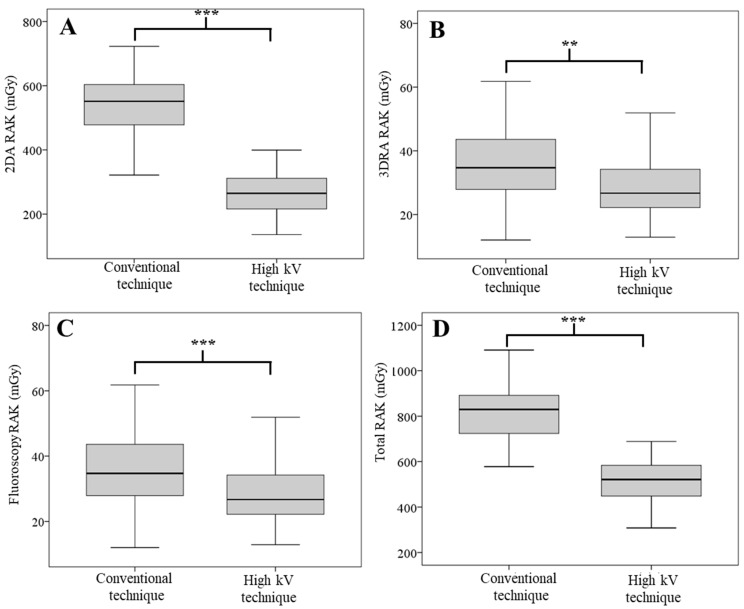
RAK from the 2DA technique (**A**), 3DRA technique (**B**), fluoroscopic technique (**C**), and Total RAK (**D**). ** and *** indicate significant differences with *p*-value < 0.01 and 0.001, respectively.

The results of the DAP and RAK from the 2DA, 3DRA, fluoroscopy, and the total procedure are shown in Figure 2 and Figure 3. Figure 2 demonstrates that the DAP of the 2DA (Figure 2A) in the high kV technique group significantly reduces compared to the conventional technique group (*p*-value < 0.001). Although the technique of 3DRA and fluoroscopy were not modified in this study, the significant reduction in DAP in both techniques (Figure 2B,C) were also observed (*p*-value < 0.01, 0.001, respectively). In addition, a significant reduction in total DAP (Figure 2D) was also observed (*p*-value < 0.001). The median 2DA DAP of the high kV technique group was 4605.37 µGy·m^2^, while this value in the conventional technique group was 9235.32 µGy·m^2^. The high kV technique reduced the 2DA DAP by up to 50.12%. In addition, the median total DAP in the high kV technique group (10,967.55 µGy·m^2^) decreased from the conventional technique group (16,198.53 µGy·m^2^) by 32.29%.

Figure 3 demonstrates the RAK, and the result shows the same trend as the DAP. The RAK of the 2DA, fluoroscopy, and total procedure (Figure 3A,C,D) in the high kV technique group was significantly reduced from the conventional technique group (*p*-value < 0.001 for all), while the RAK of the 3DRA was also found to have a significant difference (*p*-value = 0.003). The median RAK of the 2DA in the conventional technique group was 551.4 mGy, while this parameter in the high kV technique group was 264.7 mGy, which clearly signifies that the high kV technique could decrease the RAK by 51.99%. Finally, the total RAK in the high kV technique group (521.2 mGy) decreased significantly when compared to the conventional technique (829.7 mGy) by 37.18%.

### 3.3. Effects of the High kV Technique on Phantom Skin Dose

**Figure 4:**
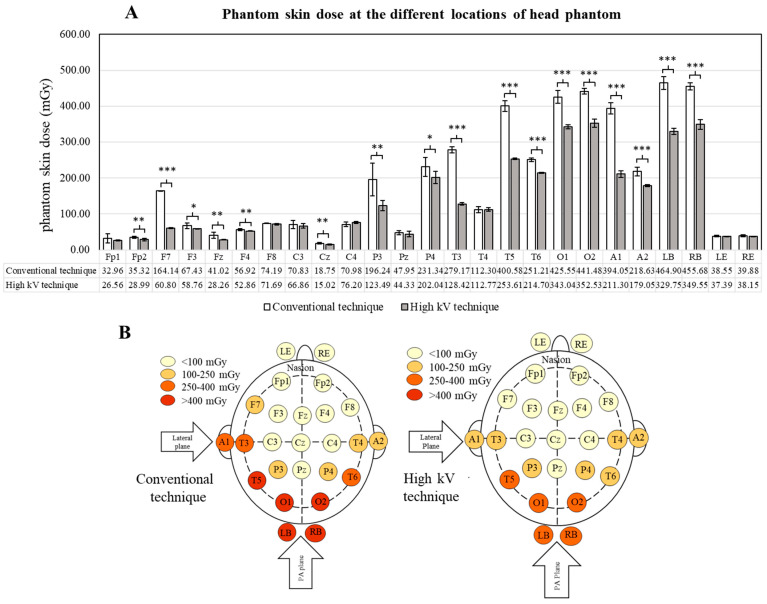
Phantom skin doses in different areas of the skull using the conventional and high kV techniques for the cerebral angiographic procedure: (**A**) bar graph demonstrates phantom skin doses, and (**B**) diagram demonstrates the distribution of radiation doses in different areas of the skull. *, **, and *** indicate a significant difference in radiation dose between the conventional and high kV techniques with *p*-value < 0.05, 0.01, and 0.001, respectively. The data on skin dose are presented as mean, while the bar graph presents the mean ± SD. A = auricle, C = central, F = frontal, Fp = prefrontal, LE = left eye, LB = back of neck on left side, O = occipital, P = parietal, RB = back of neck on right side, RE = right eye, T = temporal, and z = midline sagittal plane.

Figure 4 demonstrates the skin radiation dose at each measurement location of the head phantom. The highest phantom skin dose was observed at the back of the neck (LB and RB), followed by the occipital area (O1 and O2) and the temporal area on the left side of the head (T5). The phantom skin doses in these locations were more than 400 mGy in the conventional technique, while they were reduced to less than 400 mGy in the high kV technique, demonstrating a significant difference (*p*-value < 0.001 for all). The phantom skin doses at the location of A1, T3, and T6 in the high kV technique were found to significantly reduce when compared to the conventional technique (*p*-value < 0.001 for all). A significant difference in phantom skin dose between the conventional and high kV techniques was also observed at F7, P3, P4, and A2 (*p*-value < 0.001, 0.05, 0.05, and 0.001, respectively). The remaining locations, including Fp2, F3, Fz, F4, and Cz, where the phantom skin dose was less than 100 mGy also observed a significant difference between both techniques (*p*-value < 0.05 and 0.01 for all). In addition, it was found that the radiation doses on the eyes in the conventional technique were slightly higher than the high kV technique but did not show any significant difference (*p*-value > 0.05 for all).

### 3.4. Effects of the High kV Technique on Dose Report from the DAP Meter

The radiation dose from the cerebral angiography as measured by the DAP meter is shown in Table 3. The results showed that the DAP and RAK in the 2DA, the total DAP, and the total RAK significantly reduced in the high kV technique (*p*-value < 0.001). The other parameters did not show any significant difference between these techniques (*p*-value > 0.05). Interestingly, the 2DA DAP and the RAK in the high kV technique reduced to 47.24% and 49.02% from the conventional technique, respectively.

## 4. Discussion

This study demonstrates the effectiveness of the high kV technique in reducing the radiation dose of cerebral angiography in both the intracranial aneurysm patients and the phantom study. The high kV technique could reduce the DAP, RAK, and phantom skin radiation dose significantly.

Based on a basic knowledge, there are several factors that affect patient radiation dose during the cerebral angiographic procedure, such as the number of exposures, frame rate, additional filter thickness, focal spot size, number of images, fluoroscopic time, and pulse. Several lines of evidence have demonstrated that optimization through the modification of these factors significantly reduces radiation dose during cerebral angiography [8,10,11,15,16]. Since some factors (number of image and acquisition time) could not be controlled in this study, a statistical analysis of radiation doses from the conventional and high kV techniques using analysis of covariances (ANCOVAs) was necessary. However, the data on radiation doses had an abnormal distribution; thus, the Quade’s ANCOVA was used to analyze these data. This analysis confirmed that the reduction in radiation doses in the 2DA technique did not confound with the difference in acquisition time and number of images.

Our study shows that an increase in kV in the 2DA technique causes a reduction in mAs, which is in line with previous reports on the association of kV and mAs [17,18]. It has been reported that changes in mAs have an influence on radiation dose [19,20]. Moreover, an increase in kVp causes a reduction in the effective dose [21,22]. The results from this study showed that the use of the high kV technique in 2DA technique provided an effective reduction in radiation dose, which was in accordance with previous reports.

Apart from kV and mAs, an increase in Cu filter thickness was also observed in the high kV technique. A previous study also demonstrated that an additional Cu filter could reduce patient radiation dose during interventional fluoroscopic procedure and cerebral angiography [11,23,24]. In addition, it has been reported that Cu filter absorbs low energy photon between 20 and 50 keV, and adding a Cu filter with 0.2 mm thickness could reduce the radiation dose by 50% [25]. The automatic adding of a Cu filter in the high kV technique absorbed low energy X-ray, which is an important factor for patient skin dose. Therefore, the radiation doses from both the patients and the phantom study were reduced significantly.

Although the fluoroscopy and 3DRA technique were not modified in this study, the DAP and RAK for these techniques in the retrospective study showed a significant difference. On the other hand, the doses for the phantom study did not show any significant difference between the groups in terms of DAP and RAK. Therefore, it is possible that the size of the patients’ head in both groups might cause a difference, which in turn led to a significant difference in radiation doses for the fluoroscopy and 3DRA. However, this study was a retrospective study, and the data regarding the patients’ head size were not recorded.

The measurement of radiation dose using nanoDot™ OSLDs is appropriate and does not disturb the acquired images in all angiographic techniques. The phantom skin dose showed the same trend as the patient radiation dose in the conventional and high kV techniques. The O1, O2, RB, and LB are the centers of entrance for the radiation beam from the PA plane X-ray tube, while the A1, T3, and T5 are the centers of entrance for the radiation beam from the lateral plane X-ray. The highest skin radiation dose from this procedure does not reach the threshold dose for skin reaction [26]. The deterministic effect has a lower possibility of occurring in the case of cerebral angiography.

Since some locations on the head phantom were presented with a low dose, there were several explanations for these results. Most of the locations with a low dose were located at the exit of the radiation beam, at the rim of the field of view, and out of the field of view (Fp and Cz). In addition, it has been reported that there is a little effect of angulation on entrance skin dose measurement in diagnostic X-ray examination [27,28]. The placing of the nanoDot™ OSLDs on the surface of the head phantom at each location showed a difference in angulation to the X-ray beam, which might contribute to the difference in radiation dose measurement using the nanoDot™ OSLDs.

The eye lens doses from both techniques did not demonstrate any significant difference. It was suggested that the eye lens was at the rim of the field of view in the 2DA technique. In addition, most of the eye lens doses were obtained from the 3DRA technique, which was not modified and did not show any difference in this study. Therefore, the modification of the 2DA technique did not affect the result. There is a limitation related to the measurement of the eye lens doses. Since organ doses, particularly the eye lens dose, could not be measured with the phantom used in this study, the data set provided an approximate value that was measured at the surface of the phantom’s eyes. However, the eye lens dose of this study was far from the threshold dose, which was recommended as 0.5 Gy for low linear energy transfer radiation [26].

## 5. Conclusions

The results from this study confirmed that the high kV technique for intracranial aneurysm patients undergoing cerebral angiography provides an effective reduction of radiation dose. Further investigation of radiation dose in other interventional neuroradiology procedures, particularly embolization procedure, should be performed.

## Figures and Tables

**Table 1:** Cerebral angiographic protocol and exposure parameters of the conventional and high kV techniques in the phantom study.

Technique	Protocol		Value
Fluoroscopy	PA kV, mA		65 kV, 137.1–141.3 mA
	Lateral kV, mA		65 kV, 97.9–98 mA
	Time	PA plane	2 min
		Lateral plane	1 min
2DA	Field size	PA/Lat	32/25 cm
	SID	PA/Lat	102/94 cm
	Projection	RICA	PA view (11° cephalad) Lateral view
		LICA	
		VA	
	Frame rate and acquisition time	Mask	4 f/s 1 s
		Arterial phase	4 f/s 4 s
		Capillary phase	2 f/s 4 s
		Venous phase	1 f/s 5 s
3DRA	Field size	PA plane	42 cm
	SID	PA plane	120 cm
	Rotational arc	RICA	200 degrees
		LICA	
		VA	
	kV, mAs		71 kV, 2.25–2.46
	Number of frames/rotations		133 f/rotation
	Rotational time		5 s

2DA = 2-dimensional angiography, 3DRA = 3-dimensional rotational angiography, cm = centimeter, f = frame, f/s = frames per second, kV = kilovoltage, Lat = lateral, LICA = left internal carotid artery, min = minute, mA = milliampere, PA = posteroanterior, RICA = right internal carotid artery, s = second, SID = source to image distance, and VA = vertebral artery.

**Table 2:** The cerebral angiographic exposure parameters from the conventional and high kV techniques.

Angiographic Exposure Parameters		Conventional Technique Mean ± SD (Median)	High kV Technique Mean ± SD (Median)	*p*-Value
kV	2DA PA	77.28 ± 2.85 (77.00)	87.75 ± 2.70 (90.00)	<0.001 ^b^
	2DA Lateral	72.52 ± 1.56 (73.00)	79.59 ± 1.12 (79.00)	<0.001 ^b^
	3DRA Pre-contrast	70.00 ± 0.00 (70.00)	70.02 ± 0.13 (70.00)	0.317 ^b^
	RA Post-contrast	70.00 ± 0.00 (70.00)	70.00 ± 0.00 (70.00)	1.000 ^b^
mAs	2DA PA	19.91 ± 0.70 (19.89)	12.03 ± 1.37 (11.91)	<0.001 ^b^
	2DA Lateral	16.07 ± 3.25 (16.55)	14.98 ± 1.54 (14.98)	0.020 ^a^
	3DRA Pre-contrast	2.04 ± 0.34 (2.03)	2.13 ± 0.30 (2.13)	0.117 ^a^
	3DRA Post-contrast	2.03 ± 0.34 (2.02)	2.09 ± 0.30 (2.09)	0.305 ^a^
Cu Filter thickness (mm)	2DA PA	0.00 ± 0.00 (0.00)	0.05 ± 0.06 (0.00)	<0.001 ^b^
	2DA Lateral	0.02 ± 0.05 (0.00)	0.27 ± 0.05 (0.30)	<0.001 ^b^
2DA acquisition time (s)		14.49 ± 2.73 (15.00)	13.31 ± 2.02 (13.00)	0.005 ^b^
Total number of angiographic images		993.87 ± 13.64 (994.00)	986.52 ± 10.37 (986.00)	0.001 ^a^
Fluoroscopic time (min)		4.31 ± 4.25 (3.28)	3.30 ± 1.19 (3.03)	0.062 ^b^

^a^ Analysis using independent sample *t*-test; ^b^ Analysis using Mann–Whitney U test.

**Table 3:** The radiation dose report, including the DAP and RAK of the 2DA, 3DRA, and fluoroscopy, the total DAP, and the total RAK, from the phantom study.

Radiation Dose	Conventional Technique	High kV Technique	Percentage Reduction	*p*-Value
2DA DAP (µGy·m^2^)	12,456.97 ± 77.09	6571.80 ± 48.55	−47.24	<0.001
3DRA DAP (µGy·m^2^)	7768.97 ± 74.58	7738.23 ± 148.40	−0.40	0.765
Fluoroscopic DAP (µGy·m^2^)	977.70 ± 24.17	997.48 ± 6.87	2.02	0.244
2DA RAK (mGy)	668.07 ± 2.90	340.57 ± 13.83	−49.02	<0.001
3DRA RAK (mGy)	307.93 ± 2.95	308.67 ± 3.21	0.24	0.786
Fluoroscopic RAK	48.40 ± 1.05	48.67 ± 1.44	0.55	0.809
Total DAP (µGy·m^2^)	21,203.63 ± 126.28	15,307.52 ± 132.83	−27.81	<0.001
Total RAK (mGy)	1024.40 ± 4.37	697.90 ± 15.42	−31.87	<0.001

2DA = 2-dimensional angiography; 3DRA = 3-dimensional rotational angiography; DAP = dose area product; and RAK = reference air kerma.

## Data Availability

The data sets generated or analyzed during the study are available from the corresponding author upon reasonable request.
